# Chromosomal Locations of *mcr-1* and *bla*_CTX-M-15_ in Fluoroquinolone-Resistant *Escherichia coli* ST410

**DOI:** 10.3201/eid2209.160692

**Published:** 2016-09

**Authors:** Linda Falgenhauer, Said-Elias Waezsada, Konrad Gwozdzinski, Hiren Ghosh, Swapnil Doijad, Boyke Bunk, Cathrin Spröer, Can Imirzalioglu, Harald Seifert, Alexandra Irrgang, Jennie Fischer, Beatriz Guerra, Annemarie Käsbohrer, Jörg Overmann, Alexander Goesmann, Trinad Chakraborty

**Affiliations:** Justus Liebig University, Giessen, Germany (L. Falgenhauer, S.-E. Waezsada, K. Gwozdzinski, H. Ghosh, S. Doijad, C. Imirzalioglu, A. Goesmann, T. Chakraborty);; German Center for Infection Research, Giessen, Germany (L. Falgenhauer, S.-E. Waezsada, K. Gwozdzinski, H. Ghosh, S. Doijad, C. Imirzalioglu, T. Chakraborty);; Leibniz Institute DSMZ—German Collection of Microorganisms and Cell Cultures, Braunschweig, Germany (B. Bunk, C. Spröer, J. Overmann);; German Center for Infection Research, Braunschweig (B. Bunk, C. Spröer, J. Overmann);; University of Cologne, Cologne, Germany (H. Seifert);; German Center for Infection Research, Cologne (H. Seifert);; Federal Institute for Risk Assessment, Berlin, Germany (A. Irrgang, J. Fischer, B. Guerra, A. Käsbohrer)

**Keywords:** Colistin resistance gene mcr-1, extended-spectrum beta-lactamase, Escherichia coli, ST410, bacteria, antimicrobial resistance, fluoroquinolones, ESBL, sequence types

**To the Editor:** Recently, Yi-Yun Liu et al. reported on the discovery of *mcr-1*, a plasmidborne resistance gene mediating resistance to colistin, in isolates obtained from humans and animals (*1*). Since the original publication, *mcr-1* with or without the insertion element IS*Apl1* has been detected on plasmids of different incompatibility groups, including IncI2, IncHI2, and IncX4, and in many different countries (*1*–*3*). Because colistin is a last-resort parenteral antimicrobial drug, the transfer of *mcr-1* by conjugation or through mobilizable plasmids raises concern about the emergence of pan-resistant *Enterobacteriaceae*.

We previously described extended-spectrum β-lactamase (ESBL)–producing and carbapenemase-producing isolates obtained from livestock and a human in Germany that harbored the *mcr-1* gene (*2*). Because the transfer of *mcr-1* through the food chain is highly likely, we looked for its presence in 62 whole-genome sequenced ESBL-producing *Escherichia coli* isolates obtained during 2012–2013 from food products sampled in Germany (Technical Appendix). We detected 4 isolates harboring the *mcr-1* gene (*E. coli* RL138, RL145, RL158, and RL465) that displayed a colistin MIC of 4 mg/L (Technical Appendix Table 1). The raw sequencing reads and the assembled contigs of the *mcr-1*–positive isolates were deposited in the European Nucleotide Archive under project accession no. PRJEB13470. We conducted conjugation experiments to analyze the transferability of *mcr-1* (Technical Appendix). For all isolates except RL465, *mcr-1* was transferable to *E. coli* J53 *Az*^r^. For isolates RL138, RL145, and RL158, the *mcr-1* gene was present on IncX4 and IncHI2 plasmids (Figure, panel A; Technical Appendix Table 2). The sequence type (ST) 410 *E. coli* isolate RL465 was detected in a turkey hen meat sample from 2013 and harbored *bla*_CTX-M-15_ and *mcr-1*, a gene combination hitherto identified only in travelers from the Netherlands and children from China (*4*). Both the *bla*_CTX-M-15_ and *mcr-1* genes were not transferable, indicating that neither gene was plasmid-encoded. Examination of the genetic environment of *mcr-1* in the assembled gapped genome showed a chromosomal location for the *mcr-1* transposition unit that included an IS*Apl1* element (Figure 1, panel A; Technical Appendix Figure 1, panel A) flanked by the inverted repeats (IR-R1, IR-R2, and IR-L1). We verified the chromosomal location for the *mcr-1* gene by sequencing the genome to completion, using long-read single-molecule real-time sequencing (Pacific Biosciences, Menlo Park, CA, USA; Technical Appendix Figure 2); the resulting contigs of *E. coli* RL465 Were deposited in the European Nucleotide Archive under accession no. PRJEB14095. One copy of the IS*Apl1*-*mcr-1* transposition unit was located in the region between a predicted 4Fe-4S ferredoxin-type protein (*ydhY*) and *ldtE* (L,D-transpeptidase) (bp 2652307–2665241), and flanked on either side by a 2-bp direct repeat (CA). We observed a similar situation for the IS*Ecp1-bla*_CTX-M-15_–*orf477* transposition unit (Technical Appendix Figure 1, panel B). However, this insertion mapped to a different chromosomal location in a region encoding a defective lambdoid prophage inserted between the molybdate ABC transporter operon (*modABC*) and the biotin biosynthesis operon (*bioABCDF*) (bp 1662140–1716472). It was flanked by direct repeats (TGGTT).

**Figure:**
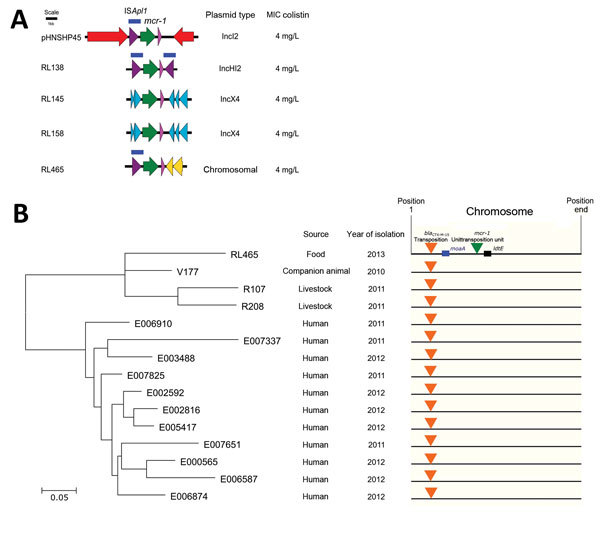
A) Genetic environment of *mcr-1* in extended-spectrum β–lactamase (ESBL)–producing food isolates including the original environment found in pHNSHP45. Colors represent gene functions and insertion sequences: purple, transposase of IS*Apl1*; dark blue, IS*Apl1* element; green, *mcr-1*; pink, hypothetical protein within the *mcr-1* transposition unit; red, light blue, yellow, flanking genes specific for the respective isolate. B) Phylogenetic tree (unrooted) of the *Escherichia coli* sequence type 410 isolates harboring the *bla*_CTX-M-15_ transposition unit in the chromosome and a schematic depiction of the location of the *mcr-1* and *bla*_CTX-M-15_ transposition unit. Isolates prefixed with the letter E represent consecutive isolates from 1 patient. Scale bar indicates nucleotide substitutions per site.

We reexamined our collection of 424 genome sequenced ESBL- and carbapenemase-encoding *E. coli* isolates, obtained during 2010–2014 (*2*), for isolates that harbored *bla*_CTX-M-15_ at a chromosomal location identical to that found in *E. coli* RL465. We detected 3 such isolates from 2010–2011 from companion animals and livestock (R107, sock swab dairy cattle farm, 2011; R208, sock swab pig fattening farm, 2011; V177, sick dog, 2010), and 11 consecutive isolates from a hemato-oncologic patient (*5*), obtained within an 11-month period during 2011–2012 (E006910, E007337, E007651, E007825, E000565, E002592, E002816, E003488, E005417, E006587, E006874) (Figure, panel B). All of these isolates were ST410 and negative for the *mcr-1* gene. Phylogenetic analysis of the core genome of these isolates with *E. coli* RL465 using the program Harvest Suite (*6*) indicated they were highly related and separated from *E. coli* V177 (the oldest isolate) by 66 (E006910, E007651) to 110 (E007337) single-nucleotide polymorphisms (core genome size 94%, representing 4.58 Mbp). Thus, our results suggest that transposition of the IS*Apl*1-*mcr-1* unit to the chromosome in *E. coli* RL465 is a later event and probably occurred after transfer of the *bla*_CTX-M-15_ allele to the distinct chromosomal location into this *E. coli* ST410 subclone.

These findings highlight 2 independent points. First, our results extend data on the mobility of IS*Apl*1-*mcr-1* to a chromosomal location and reveal a new dimension in the transmissible nature of *mcr-1* in colistin-resistant *Enterobacteriaceae* isolates and their ecology. Second, clonal isolates of ST410 have been isolated from diverse environments, livestock, companion animals, and humans and, as we demonstrate here, in turkey hen meat (*7*,*8*). Thus, the simultaneous spread of the *mcr-1* and *bla*_CTX-M-15_ genes mediated by a single bacterial clone is real and suggests that *mcr-1* is already present in the diverse reservoirs inhabited by these isolates.

Technical Appendix Collection of isolates; antimicrobial susceptibility testing; whole-genome sequencing; in silico analyses; conjugation experiments; depiction of the minimal inhibitory concentration of the *mcr-1*–encoding and extended-spectrum β-lactamase (ESBL)–producing isolates from retail food; characteristics of the *mcr-1*–encoding ESBL-producing *Escherichia coli* isolates from retail meat; genetic environments of the chromosomally located antimicrobial resistance genes; schematic depiction of the chromosome, its IncFII/FIB plasmid, and the 2 phage elements in *E. coli* RL465.
