# Supplementary material for: Chromosomal Locations of mcr-1 and blaCTX-M-15 in Fluoroquinolone-Resistant Escherichia coli ST410
Source: Emerg Infect Dis. 2016 Sep;22(9):1689–91. doi: 10.3201/eid2209.160692 (PMC4994348; doi:10.3201/eid2209.160692)
Supplement: Technical Appendix — Collection of isolates; antimicrobial susceptibility testing; whole-genome sequencing; in silico analyses; conjugation experiments; depiction of the minimal inhibitory concentration of the mcr-1–encoding and extended-spectrum β-lactamase (ESBL)–producing isolates from retail food; characteristics of the mcr-1–encoding ESBL-producing Escherichia coli isolates from retail meat; genetic environments of the chromosomally located antimicrobial resistance genes; schematic depiction of the chromosome, its IncFII/FIB plasmid, and the 2 phage elements in E. coli RL465. [file 16-0692-Techapp-s1.pdf]

Article DOI: http://dx.doi.org/10.3201/eid2209.160692

Chromosomal Locations of *mcr-1* and *bla*$_{CTX-M-15}$ in Fluoroquinolone-Resistant *Escherichia coli* ST410

Technical Appendix

Collection of Isolates

Retail food samples originating from cattle, swine, and poultry (meat and milk) were sampled by food inspectors in 4 different regions of Germany during May 2012–April 2013. All samples were unrelated to each other. No information was recorded about the country from which the animals originated. Extended-spectrum β-lactamases (ESBL)–producing isolates were selected by using MacConkey agar plates supplemented with 1 mg/L cefotaxime. A subset of 62 *Escherichia coli* isolates was analyzed by using whole-genome sequencing.

Antimicrobial Susceptibility Testing

Antimicrobial susceptibility tests were performed by using VITEK 2 (AST-card: N248; bioMérieux, Nürtingen, Germany). Colistin susceptibility testing was performed by using broth microdilution according to European Union Commission Implementing Decision 2013/652/EU (http://eur-lex.europa.eu/legal-content/EN/TXT/?uri = CELEX:32013D0652) (using EUVSEC Sensititer plates, Trek Diagnostic systems, Thermo Fischer Scientific, Dreieich, Germany). The results were interpreted according to the European Committee on Antimicrobial Susceptibility Testing (2015) and Clinical and Laboratory Standards Institute (2015) guidelines (*1*,*2*).

Whole-Genome Sequencing

Whole-genome DNA was isolated from overnight cultures by using the Purelink Genome DNA Mini kit (Invitrogen, Darmstadt, Germany). The sequencing library was produced by using Illumina Nextera XT Kit and sequenced on a MiSeq instrument (Illumina, San Diego, CA,

USA), with 2 × 300 read length. The average read length accounted for 180 nt with an average coverage of 51×. Raw reads were assembled by using SPAdes (v. 3.0) (*3*). To confirm the chromosomal location of *mcr-1* in *E. coli* RL465, long-read single-molecule real-time (SMRT) sequencing was performed. For this, DNA was isolated by using the method described by Pitcher et al. (*4*). A SMRTbell template library was prepared according to the instructions, following the Procedure & Checklist—10 kb Template Preparation Using BluePippin Size-Selection System. Briefly, for preparation of 15-kb libraries 8 μg genomic DNA was sheared by using g-tubes (Covaris, Woburn, MA, USA) according to the manufacturer´s instructions. DNA was end-repaired and ligated overnight to hairpin adapters applying components from the DNA/Polymerase Binding Kit P6 (Pacific Biosciences, Menlo Park, CA, USA). Reactions were conducted according to the manufacturer´s instructions. BluePippin Size-Selection to 4 kb was performed according to the manufacturer´s instructions (Sage Science, Beverly, MA, USA). Conditions for annealing of sequencing primers and binding of polymerase to purified SMRTbell template were assessed with the Calculator in RS Remote (Pacific Biosciences). SMRT sequencing was conducted on the PacBio RSII (Pacific Biosciences) taking one 240-min movie for a single SMRT cell. We assembled PacBio reads using RS_ HGAP_ Assembly.3 protocol included in the SMRT Portal 2.3.0. The number of reads from PacBio sequencing accounted for 93,593 with a mean read length of 12,057 nt. To obtain a high-quality genome sequence, we mapped paired-end reads from Illumina sequencing using Burrows–Wheeler Aligner (*5*). The chromosome displayed a size of 4,894,900 bp (167× coverage). One plasmid of 157,187 bp (57× coverage), and 2 phage-like elements 89,746 bp, (Element 1, 39× coverage, circular), and 61,544 bp (Element 2, 234× coverage, linear) were detected.

In Silico Analyses

We identified resistance genes using ResFinder (*6*), virulence genes using VirulenceFinder (*7*), plasmid incompatibility groups and plasmid multilocus sequence typing with PlasmidFinder and pMLST (*8*), and multilocus sequence types using MLST 1.8, according to the scheme of Wirth et al. (*9*,*10*). The genetic environment of *mcr-1* and $bla_{CTX\text{-}M\text{-}15}$ was identified using blastn and ISFinder (*11*,*12*). Annotation of the *E. coli* RL465 genome and extrachromosomal units was performed using RAST (*13*). To identify phages, we used the program PHAST (*14*).

Conjugation Experiments

We conducted conjugation experiments at 37°C or at ambient temperatures as described previously (*15*) using *E. coli* J53 *Az*^{r} as a recipient and 2 mg/L colistinsulfate and 200 mg/L sodiumazide as selective agents. Replicon typing of the transconjugants was performed as described in the literature (*16*,*17*).

References

1. European Committee on Antimicrobial Susceptibility Testing. Breakpoint tables for interpretation of MICs and zone diameters. Version 5.0, 2015 [cited 2015 May 3]. http://www.eucast.org

2. Clinical and Laboratory Standards Institute. Performance standards for antimicrobial susceptibility testing. Twenty-fifth informational supplement. CLSI document M100–S25. Wayne (PA): The Institute; 2015.

3. Bankevich A, Nurk S, Antipov D, Gurevich AA, Dvorkin M, Kulikov AS, et al. SPAdes: a new genome assembly algorithm and its applications to single-cell sequencing. J Comput Biol. 2012;19:455–77. PubMed http://dx.doi.org/10.1089/cmb.2012.0021

4. Pitcher DG, Saunders NA, Owen RJ. Rapid extraction of bacterial genomic DNA with guanidium thiocyanate. Lett Appl Microbiol. 1989;8:151–6. http://dx.doi.org/10.1111/j.1472-765X.1989.tb00262.x.

5. Li H, Durbin R. Fast and accurate short read alignment with Burrows-Wheeler transform. Bioinformatics. 2009;25:1754–60. PubMed http://dx.doi.org/10.1093/bioinformatics/btp324

6. Zankari E, Hasman H, Cosentino S, Vestergaard M, Rasmussen S, Lund O, et al. Identification of acquired antimicrobial resistance genes. J Antimicrob Chemother. 2012;67:2640–4. PubMed http://dx.doi.org/10.1093/jac/dks261

7. Joensen KG, Scheutz F, Lund O, Hasman H, Kaas RS, Nielsen EM, et al. Real-time whole-genome sequencing for routine typing, surveillance, and outbreak detection of verotoxigenic *Escherichia coli*. J Clin Microbiol. 2014;52:1501–10. PubMed http://dx.doi.org/10.1128/JCM.03617-13

8. Carattoli A, Zankari E, García-Fernández A, Voldby Larsen M, Lund O, Villa L, et al. In silico detection and typing of plasmids using PlasmidFinder and plasmid multilocus sequence typing. Antimicrob Agents Chemother. 2014;58:3895–903. PubMed http://dx.doi.org/10.1128/AAC.02412-14

9. Zankari E, Hasman H, Kaas RS, Seyfarth AM, Agersø Y, Lund O, et al. Genotyping using whole-genome sequencing is a realistic alternative to surveillance based on phenotypic antimicrobial susceptibility testing. J Antimicrob Chemother. 2013;68:771–7. PubMed http://dx.doi.org/10.1093/jac/dks496

10. Wirth T, Falush D, Lan R, Colles F, Mensa P, Wieler LH, et al. Sex and virulence in *Escherichia coli*: an evolutionary perspective. Mol Microbiol. 2006;60:1136–51. PubMed http://dx.doi.org/10.1111/j.1365-2958.2006.05172.x

11. Altschul SF, Gish W, Miller W, Myers EW, Lipman DJ. Basic local alignment search tool. J Mol Biol. 1990;215:403–10. PubMed http://dx.doi.org/10.1016/S0022-2836(05)80360-2

12. Siguier P, Perochon J, Lestrade L, Mahillon J, Chandler M. ISfinder: the reference centre for bacterial insertion sequences. Nucleic Acids Res. 2006;34:D32–6. PubMed http://dx.doi.org/10.1093/nar/gkj014

13. Aziz RK, Bartels D, Best AA, DeJongh M, Disz T, Edwards RA, et al. The RAST Server: rapid annotations using subsystems technology. BMC Genomics. 2008;9:75. PubMed http://dx.doi.org/10.1186/1471-2164-9-75

14. Zhou Y, Liang Y, Lynch KH, Dennis JJ, Wishart DS. PHAST: a fast phage search tool. Nucleic Acids Res. 2011;39(suppl):W347-5. PubMed2 http://dx.doi.org/10.1093/nar/gkr485

15. Falgenhauer L, Waezsada S-E, Yao Y, Imirzalioglu C, Käsbohrer A, Roesler U, et al.; RESET consortium. Colistin resistance gene *mcr-1* in extended-spectrum β-lactamase-producing and carbapenemase-producing gram-negative bacteria in Germany. Lancet Infect Dis. 2016;16:282–3. PubMed http://dx.doi.org/10.1016/S1473-3099(16)00009-8

16. Carattoli A, Bertini A, Villa L, Falbo V, Hopkins KL, Threlfall EJ. Identification of plasmids by PCR-based replicon typing. J Microbiol Methods. 2005;63:219–28. PubMed http://dx.doi.org/10.1016/j.mimet.2005.03.018

17. Johnson TJ, Bielak EM, Fortini D, Hansen LH, Hasman H, Debroy C, et al. Expansion of the IncX plasmid family for improved identification and typing of novel plasmids in drug-resistant *Enterobacteriaceae*. Plasmid. 2012;68:43–50. PubMed http://dx.doi.org/10.1016/j.plasmid.2012.03.001

Technical Appendix Table 1. Depiction of the MIC of the *mcr-1*-encoding and extended-spectrum β-lactamase ESBL–producing isolates from retail food*

Antimicrobial drug class, drug	RL138		RL145		RL158		RL465	
	MIC	Interpretation	MIC	Interpretation	MIC	Interpretation	MIC	Interpretation
Aminoglycosides								
Amikacin	≤2	S	≤2	S	≤2	S	≤2	S
Gentamicin	≤1	S	≤1	S	≤1	S	≤1	S
Tobramycin	≤1	S	≤1	S	≤1	S	≤1	S
Penicillins								
Ampicillin	≥32	R	≥32	R	≥32	R	≥32	R
Ampicillin/Sulbactam	16	R	≥32	R	16	R	16	R
Piperacillin	≥128	R	≥128	R	≥128	R	≥128	R
Piperacillin/Tazobactam	≤4	S	8	S	≤4	S	≤4	S
Carbapenems								
Ertapenem	≤0.5	S	≤0.5	S	≤0.5	S	≤0.5	S
Imipenem	≤0.25	S	≤0.25	S	≤0.25	S	≤0.25	S
Meropenem	≤0.25	S	≤0.25	S	≤0.25	S	≤0.25	S
Cephalosporins								
Cefepime	2	I	≤1	S	2	I	2	I
Cefotaxime	8	R	8	R	8	R	32	R
Cefpodoxime	≥8	R	≥8	R	≥8	R	≥8	R
Ceftazidime	≤1	S	16	R	≤1	S	4	R
Cefuroxime	≥64	R	≥64	R	≥64	R	≥64	R
Fluoroquinolones								
Ciprofloxacin	≥4	R	≤0.25	S	≤0.25	S	≥4	R
Moxifloxacin	≥8	R	≤0.25	S	≤0.25	S	≥8	R
Miscellaneous agents								
Fosfomycin	≤16	S	≤16	S	≤16	S	≤16	S
Trimethoprim/ sulfamethoxazole	≥320	R	≤20	S	≥320	R	≥320	R
Monobactams: Aztreonam	≤1	S	2	I	2	I	16	R
Tetracyclines								
Tetracycline	≥16	R	≥16	R	≤1	R	≥16	R
Tigecycline	≤0.5	S	≤0.5	S	≤0.5	S	≤0.5	S

*The MIC results were interpreted according to the European Committee on Antimicrobial Susceptibility Testing (2015) and the Clinical and Laboratory Standards Institute (2015) guidelines (*1,2*). MIC, minimal inhibitory concentration; R, resistant; S, susceptible.

Technical Appendix Table 2. Characteristics of the *mcr-1*-encoding ESBL-producing *Escherichia coli* isolates from retail meat*

Variable	Characteristic			
Name	RL138	RL145	RL158	RL465
Species	*E. coli*	*E. coli*	*E. coli*	*E. coli*
Sequence type	ST602	ST88	ST357	ST410
Year of isolation	2012	2012	2012	2013
Colistin MIC	4 mg/L	4 mg/L	4 mg/L	4 mg/L
Source	Chicken breast	Minced meat, beef	Turkey hen Schnitzel	Turkey hen meat
Antimicrobial resistance genes	*aadA1, aadA2-like, bla*$_{\text{CTX-M-14}}$, *catA1-like, cmlA1-like, dfrA1, mcr-1, strA-like, strB, sul1, sul3, tet(A)*	*aadA1-like, aph(3′)-Ia-like, bla*$_{\text{OXA-1}}$, *bla*$_{\text{TEM-52C}}$, *mcr-1, sul1, tet(B)*	*aadA1, bla*$_{\text{CTX-M-1}}$, *dfrA1, mcr-1, sul1-like, sul2*	*aadA1*, *aph(3′)-Ia*, *bla*$_{\text{CTX-M-15}}$, *bla*$_{\text{TEM-1B}}$, *dfrA1, mcr-1, strA-like, strB-like, sul2, sul3, tet(A)-like*
Virulence genes	*iroN, iss, lpfA, mchF, tsh*	*iha, iroN, iss, lpfA, mchF, mcmA*	*iroN, iss, mchF*	*cma, iroN, iss, lpfA*
Plasmid incompatibility groups	IncHI2, IncFIC(FII), IncHI2A, IncI1, IncFIB(AP001918), IncFIA, IncP, Col(MG828)	IncFIB(AP001918), IncI1, IncFIC(FII), IncI2, IncX4, ColRNAI	IncFIC(FII), IncFIA, IncI1, IncFIB(AP001918), ColRNAI, IncX4, Col(MG828)	IncFIB(AP001918), IncFII(pCoo), Col156, ColRNAI
pMLST	IncHI2[ST-4], IncI1(unknown sequence type), IncF[F18:A6*:B1]	IncI1[ST-36], IncF[F18:A-:B1]	IncF[F18:A6*:B1], IncI1[ST-7]	IncF [F16:A-:B1]
mcr-1 location	IncHI2 plasmid	IncX4 plasmid	IncX4 plasmid	Chromosome

*MIC, minimal inhibitory concentration; pMLST, plasmid multilocus sequence type.

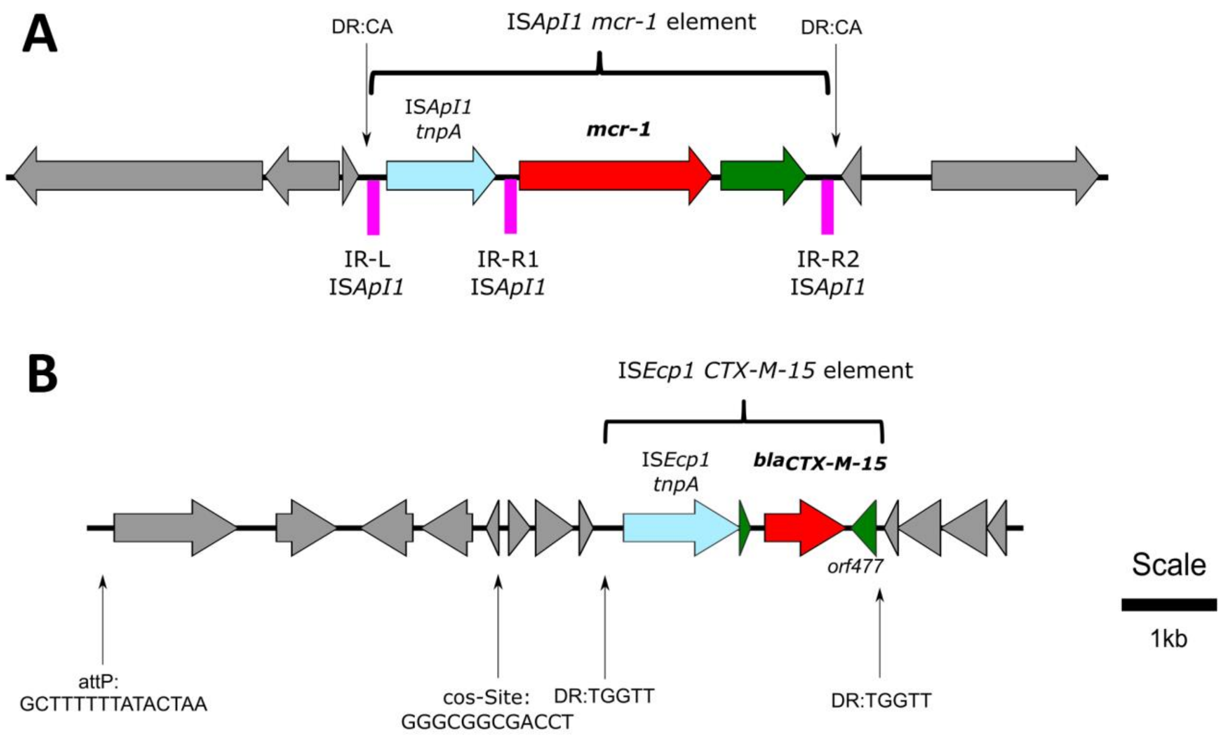

Technical Appendix Figure 1. Genetic environments of the chromosomally located antimicrobial resistance genes (A) *mcr-1* and (B) *bla*$_{CTX-M-15}$ of the *Escherichia coli* isolate RL465. *attR*, right phage attachment site; DR, direct repeat; cos site, cohesive end sequence of prophage; IR, inverted repeats. Genes marked in red display an antimicrobial resistance gene; in light blue transposase genes from the transposition units, green, other genes in the transposition units, pink bars depict the presence of the inverted repeats of IS*ApI1.* Unrelated flanking genes are shaded gray.

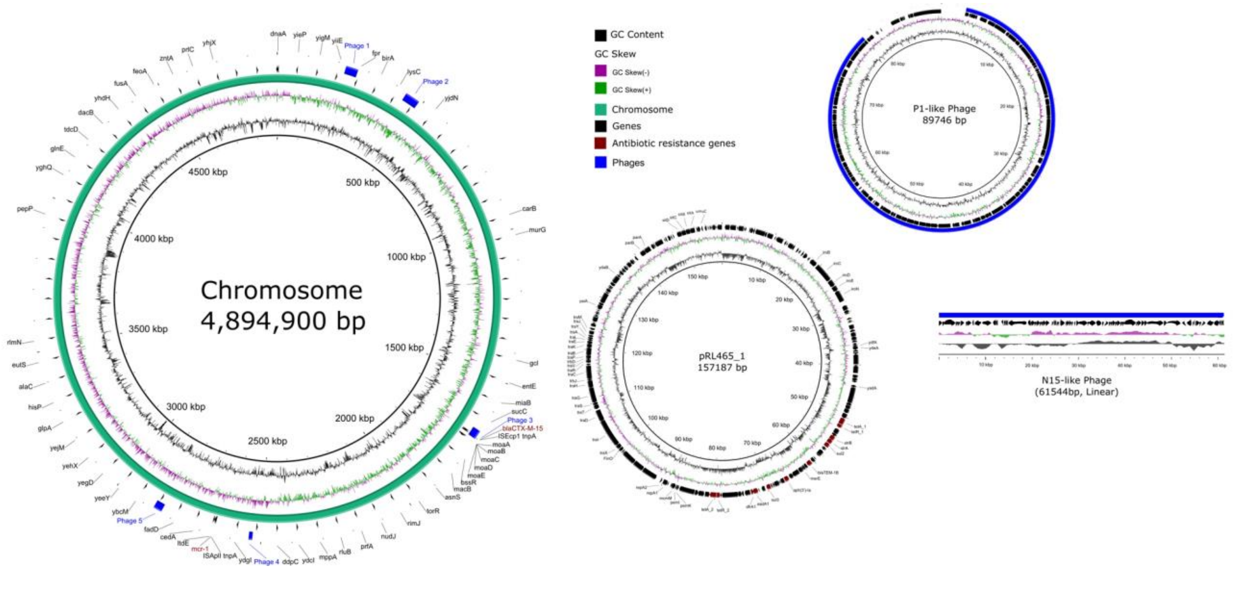

Technical Appendix Figure 2. Schematic depiction of the chromosome, its IncFII/FIB plasmid and the 2 phage elements in *Escherichia coli* RL465.
